# Minimally Invasive Porcine Model for Chronic Thromboembolic Pulmonary Hypertension

**DOI:** 10.1002/pul2.70344

**Published:** 2026-07-14

**Authors:** Bianca Battilana, Christian T. Stoeck, Fabien Robert, Ömer Senbaklavaci, Miriam Weisskopf, Isabelle Opitz

**Affiliations:** ^1^ Department of Thoracic Surgery University Hospital Zurich Zurich Switzerland; ^2^ PhD Program Biomedicine University of Zurich Zurich Switzerland; ^3^ Center for Preclinical Development University Hospital Zurich Zurich Switzerland; ^4^ Faculty of Medicine University of Zurich Zurich Switzerland

**Keywords:** CTEPH, in vivo model, microvasculopathy, porcine model, pulmonary vascular disease

## Abstract

To elucidate the complex pathophysiology of chronic thromboembolic pulmonary hypertension (CTEPH), a disease associated with bilateral fibrotic obstructions of the pulmonary arteries (PA) and microvascular changes, research relies on animal models, which often depend on invasive techniques, including open surgery. As animal welfare is a major priority for the future of experimental research, and in accordance with the principles of the 3Rs (Replacement, Reduction, and Refinement), this project aimed to develop a fully minimally invasive porcine CTEPH model. In five female large white pigs (50 kg, 3–4 months), an intravascular plug was inserted into the left PA. The right lower lobe artery was embolized weekly for 5 weeks with non‐resolving n‐butyl‐2‐cyanoacrylate‐glue. Magnetic resonance imaging (MRI) was performed at each intervention. At week 6, plasma molecular and macro‐ and microscopic analyses were performed. Implantation of the intravascular plug was successful in all animals, without any residual perfusion seen in MRI angiography. Significant increases in mean pulmonary artery pressure (mPAP) (*p* = 0.005) and mean total pulmonary resistance (TPR) (*p* = 0.043) were observed. Right ventricle dimensions were significantly increased in all animals. Macroscopically, the left lung developed hypertrophy of bronchial arteries and the right upper lobe overperfusion. Histologically, microvascular wall thickness was increased in both the over‐perfused and ischemic territories. Plasma molecular analysis revealed elevated circulating endothelin‐1 (*p* < 0.0001) and reduced nitric oxide metabolites (*p* = 0.0007). In conclusion, this study establishes a fully minimally invasive refinement of a previously described porcine CTEPH model, including relevant hemodynamic, morphologic, molecular and imaging features of CTEPH while increasing welfare for experimental animals.

## Graphical abstract

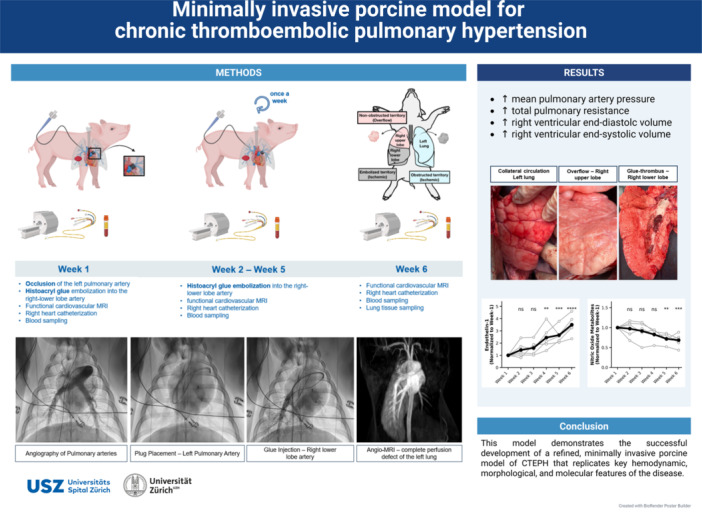

**Abbreviations**
BW: Body Weight
CMR: Cardiovascular Magnetic Resonance Imaging
CTEPH: Chronic Thromboembolic Pulmonary Hypertension
LV: Left Ventricle
LVCO: Left Ventricular Cardiac Output
LVEDV: Left Ventricular End‐Diastolic Volume
LVEF: Left Ventricular Ejection Fraction
LVESV: Left Ventricular End‐Systolic Volume
LVSV: Left Ventricular Stroke Volume
mPAP: Mean Pulmonary Artery Pressure
MRI: Magnetic Resonance Imaging
PA: Pulmonary Artery
PAWP: Pulmonary Artery Wedge Pressure
PEA: Pulmonary Endarterectomy
PH: Pulmonary Hypertension
PVR: Pulmonary Vascular Resistance
RV: Right Ventricle
RVEDV: Right Ventricular End‐Diastolic Volume
RVEF: Right Ventricular Ejection Fraction
RVESV: Right Ventricular End‐Systolic Volume
RVSV: Right Ventricular Stroke Volume
TPR: Total Pulmonary Resistance
WU: Wood Units

## 1 Introduction

Chronic thromboembolic pulmonary hypertension (CTEPH)—a rare precapillary form of pulmonary hypertension (PH)—is a potentially fatal disease often associated with preceding acute pulmonary embolism, which develops into a bilateral chronic fibrotic obstruction of the pulmonary arteries and microvascular changes in the arterial bed [1, 2]. If left untreated, CTEPH can result in right heart failure and death [2]. By removing the fibrotic obstructions, pulmonary endarterectomy (PEA) is the only curative and gold standard treatment for CTEPH [2]. The underlying pathophysiology of CTEPH is complex and currently poorly understood. Therefore, improving knowledge of this disease is vital for future surgical and medical management.

For this purpose, several large and small animal models have been developed over the past decades. Small‐animal models offer practical and ethical advantages but do not fully reproduce the spatial and vascular heterogeneity of human CTEPH [3, 4, 5, 6]. In contrast, large‐animal models more closely mimic human cardiopulmonary anatomy, pulmonary hemodynamics, and right ventricular adaptation. They therefore remain essential translational platforms for CTEPH research [3, 4, 5, 6]. A well‐established porcine CTEPH model was developed in 2013 and reproduced major features of the human disease [7]. This model combined surgical ligation of the left pulmonary artery through midline sternotomy with repeated embolization of the right lower lobe using non‐resorbable glue. By creating both obstructed territories exposed to chronic ischemia and non‐obstructed territories exposed to overflow after blood flow redistribution, the model induced pulmonary hemodynamic impairment, remodeling and wall thickening of small pulmonary arteries and alterations in circulating vasoactive factors. This original model has been widely used in translational and physiological studies and has substantially improved the understanding of vascular remodeling in different pulmonary vascular territories, molecular signaling pathways, and right ventricular adaptation in CTEPH [7, 8, 9]. Despite its physiological relevance, this model requires an invasive surgical procedure, which raises important animal welfare concerns and may limit broader implementation. Improving animal welfare is now a central priority in translational research and is aligned with the principles of the 3Rs (Replacement, Reduction, and Refinement) [10]. In this context, refinement of established large‐animal models is essential, provided that their physiological relevance is preserved. Furthermore, the testing of subsequent invasive procedures may be facilitated if the model establishment is kept minimally invasive.

The aim of the present study was therefore to refine the previously established porcine CTEPH model by replacing surgical left pulmonary artery ligation with transcatheter vascular plug occlusion. We hypothesized that this minimally invasive approach would reproduce the major hemodynamic, morphological, and molecular features of CTEPH while reducing procedural invasiveness, improving animal welfare, and facilitating broader use of the model for future translational research.

## 2 Methods

### 2.1 Ethical Approval

The study was conducted in accordance with the Principles of Laboratory Animal Care (Art. 18 Animal protection law) and was approved by the local veterinary ethics committee for animal experimentation (Vetamt Cantonal Nr. ZH130/2024, National Nr. 36904).

### 2.2 Experimental Design

**Figure 1:**
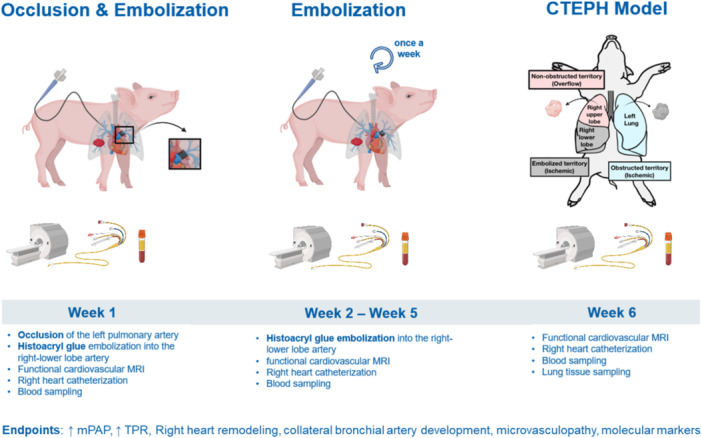
Experimental design for the in vivo porcine CTEPH model establishment. Week 1 includes the Occlusion of the left pulmonary artery and embolization of the right lower lobe artery with non‐resolvable glue. The glue embolization is repeated once a week during Week 2 to Week 5. At each time point, blood is collected. Week 6 marks the time of the last hemodynamic and imaging assessments, sacrifice of the animal and lung tissue collection. Created in BioRender. Battilana, B. (2026) https://BioRender.com/eotb6j4.

Female Swiss large white pigs (Schweizer–Edelschwein; body weight (BW) 50 kg, approximately 3–4 months of age, *n* = 5) were studied. The CTEPH model establishment spans over a period of 6 weeks. After arrival, all animals are acclimatized for 14 days. In Week 1, the CTEPH model is initiated by occlusion of the left pulmonary artery with an intravascular plug and embolization of the right lower lobe artery with non‐resolvable glue, mimicking the non‐resolving thromboembolisms found in CTEPH patients. The embolization with this non‐resolvable glue is repeated once a week during Weeks 2 to 5 (Figure 1). One week after the final embolization (Week 6), the final imaging and hemodynamic assessments and finally the sacrifice of the animals were performed. Fluoroscopic angiography, cardiovascular magnetic resonance imaging (CMR) and hemodynamic assessments with a Swan‐Ganz Catheter were performed, and plasma samples were obtained weekly prior to intervention. After the sacrifice (Week 6) macro‐ and microscopic assessment of the lungs and pulmonary arteries were performed.

### 2.3 Transcatheter Procedures

For each intervention (Week 1 to Week 5), the pigs were anesthetized with i.m. Ketamine hydrochloride (15 mg/kg BW), Azaperone (2 mg/kg BW), Atropine (0.05 mg/kg BW) and before intubation anesthesia was deepened with a i.v. Propofol bolus (1–2 mg/kg BW). Following induction of general anesthesia, a 7.5‐Fr, 2‐lumen Swan‐Ganz pulmonary artery catheter (Edwards Lifesciences, Irvine, CA) was placed through a jugular vein access sheath (8Fr) and advanced into the main pulmonary artery to monitor pulmonary artery pressure and, if possible, measure the pulmonary artery wedge pressure (PAWP).

**Figure 2:**
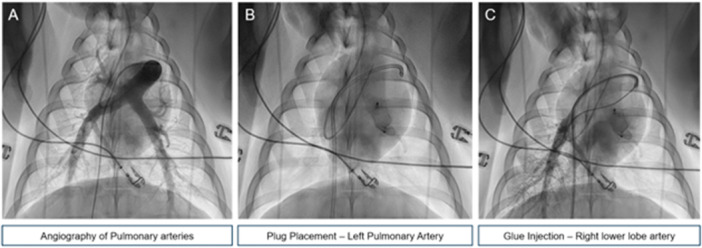
Fluoroscopic angiography showing the pulmonary vascular tree outlined by contrast (A) to enable measurement of the left pulmonary artery diameter and correct localization for transcatheter intravascular plug placement into the left pulmonary artery (B) and separate contrast injection into the right lower lobe artery for the level assessment of the histoacryl‐glue injection into the right lower lobe artery (C).

On Week 1, through a femoral vein access sheath (14 Fr) a pigtail catheter was advanced into the main pulmonary artery. A fluoroscopic contrast angiography was performed to assess the size of the left pulmonary artery. An intravascular plug (Amplatzer™ Vascular Plug II, Abott) was inserted via the femoral venous access port into the left pulmonary artery and deployed (Figure 2). Complete occlusion of the left pulmonary artery was verified by fluoroscopic angiography. Subsequently, a catheter was advanced into the right lower lobe artery for thrombo‐embolization with n‐butyl‐2‐cyanoacrylate glue (Histoacryl®; B. Braun, Melsungen, Germany). The glue‐embolization was repeated once a week during Weeks 2 to 5. The amount of Histoacryl injected did not exceed 1 ml/week and depended on hemodynamic and respiratory tolerance of the pigs. Embolization was stopped when there was an immediate increase in mPAP > 5 mmHg and/or if there were hemodynamic instabilities and/or oxygen saturation dropped < 95% during the intervention.

### 2.4 Hemodynamic Measurements

Systemic and pulmonary hemodynamic variables, blood gases and oxygen saturation were continuously measured during the interventions. Mean pulmonary artery pressure (mPAP), cardiac output (CO), and, whenever technically feasible, pulmonary artery wedge pressure (PAWP) were recorded at each intervention. Total pulmonary resistance (TPR) was calculated as (mPAP/CO); TPR is expressed in Wood Units (WU). Pulmonary vascular resistance (PVR) was calculated as (mPAP‐PAWP)/CO only for measurements with a reliable PAWP tracing. Because PAWP could not be obtained consistently in all animals and at all interventions, longitudinal analyses were performed for mPAP and TPR in the full cohort and for PAWP/PVR in the subset of available measurements.

### 2.5 Cardiovascular MRI Assessment

Following induction of general anesthesia and prior to each intervention, the animals underwent a functional CMR for assessment of right heart function and anatomy on a clinical 1.5 T system (AmbitionX, Philips Healthcare, Best, the Netherlands). Since the left ventricle was not expected to be influenced by the experimental intervention and was assumed to reflect physiological cardiac growth associated with body growth, changes in left‐ventricular volume were used as an internal reference to normalize right‐ventricular end‐diastolic and end‐systolic volumes. To this end, multi‐slice balanced steady state free precession cine acquisitions of the transverse as well as short axis orientation were performed covering the entire heart (spatial resolution: 1.7 × 1.7 mm^2^, slice thickness 8 mm, TR/TE 3.2/1.6 ms, 25 heart phases, retrospective ECG gating).

### 2.6 Light Microscopy and Morphometry of Harvested Lungs

As CTEPH development is associated with the formation of secondary microvasculopathy, pulmonary artery wall thickness was quantified on αSMA immunohistochemical staining by light microscopy. Measurements were performed in the ischemic obstructed (left upper lobe) and overflow non‐obstructed (right upper lobe) territories and compared with control tissues. For that purpose, 1 week after the final glue‐embolization, the fully inflated lungs were harvested in toto, and samples were taken from the right and left upper lobes. The samples were fixed in formalin buffer and embedded in paraffin. As a control, *n* = 6 formalin‐fixed paraffin‐embedded samples from *n* = 3 experimental pigs without any pulmonary or cardiovascular disease (both right and left lungs for each pig) were obtained through a collaborative initiative within the Center for Preclinical Development, Zurich, Switzerland. Lung sections were stained for alpha smooth muscle actin (αSMA). Wall thickness was calculated in alpha‐SMA‐positive pulmonary arteries as [(2 × medial wall thickness/external diameter) × 100] and expressed as a percentage. To increase morphometric granularity, arteries were analyzed separately in two predefined diameter categories: < 50 µm and 50–200 µm.

### 2.7 Molecular Pathways – ELISA and Colorimetric Assays

For pharmacological treatment of CTEPH in humans, three main molecular pathways are currently targeted: the endothelin‐1 pathway, the nitric oxide pathway, and the prostacyclin pathway [11]. To investigate the involvement of these pathways in the refined CTEPH model, plasma samples were collected and analyzed weekly from pigs between Week 1 (CTEPH induction) and Week 6 (sacrifice). Since nitric oxide and prostacyclin are unstable in plasma, their stable metabolites, nitrites/nitrates for nitric oxide and 6‐keto‐PGF1α for prostacyclin, were quantified instead. Blood was collected from the jugular vein into EDTA tubes. Plasma was processed according to the manufacturer's instructions and stored at −20°C for further use. Circulating endothelin‐1 levels were quantified using an ELISA kit (Bio‐Techne, catalog number DET100). Nitrite/nitrate levels, metabolites of nitric oxide, were quantified using a colorimetric assay (Sigma–Aldrich, catalog number 23479). 6‐keto‐PGF1α levels, a metabolite of prostacyclin, were quantified by ELISA (Abcam, catalog number ab133023). For each animal, results were normalized to Week 1 values to determine fold changes relative to baseline.

### 2.8 Statistical Analysis

For the descriptive data analysis and presentation, the total number, percentage and mean ± standard deviation (SD) were used. The p‐values for the hemodynamic parameters and heart volumes were calculated with a paired *t*‐test, and the significance level was defined at *p* < 0.05. The data was managed using Microsoft Excel 2016. Graphs were created using RStudio (2020) RStudio: Integrated Development for R or GraphPad Prism version 10. ELISA and colorimetric assays performed on pig plasma from Week 1 to Week 6 were analyzed using one‐way repeated measures ANOVA. For the histological analysis, wall thickness was compared between CTEPH territories (right upper and left upper lobes) and control tissue separately for arteries < 50 µm and 50–200 µm using one‐way ANOVA.

## 3 Results

The implantation of the intravascular plug was successful in all animals. The plug completely occluded the left pulmonary artery, and the following fluoroscopic angiography and MRI evaluations revealed no residual perfusion of the left lung after intravascular plug placement (Figure 2). None of the experimental pigs died prior to the scheduled sacrifice at Week 6, and no major adverse events occurred.

### 3.1 Hemodynamic Assessments

**Figure 3:**
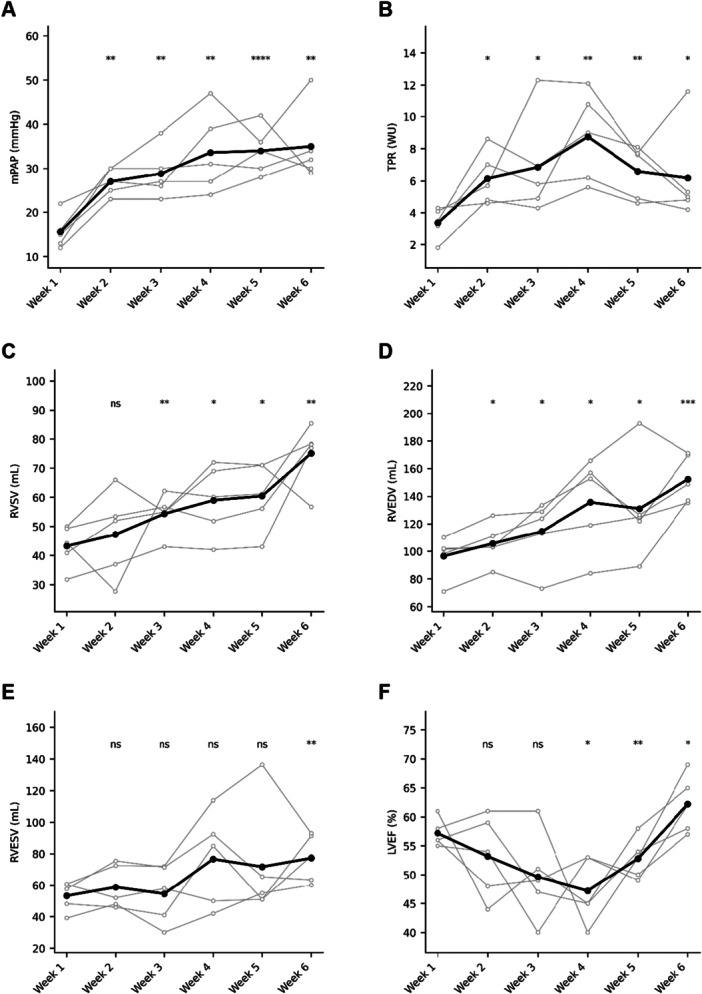
Hemodynamic assessments from Week 1 to Week 6. Significant increases in mean pulmonary artery pressure (mPAP, *p* = 0.005) (A), total pulmonary resistance (TPR, *p* = 0.04) (B), Right ventricle stroke volume (RVSV, *p* = 0.0017) (C), Right ventricle enddiastolic volume (RVEDV, *p* = 0.00061) (D), Right ventricle endsystolic volume (RVESV, *p* = 0.0098) (E) and Left ventricle ejection fraction (LVEF, *p* = 0.0256) (F) between Week 1 and Week 6 of the experimental period were observed. Individual changes are shown from Week 1 to Week 6, with gray lines representing individual pigs and the thicker black line representing the group mean. Statistical comparisons were performed for each week versus Week 1 using paired t‐tests. Significance annotations indicate ns, not significant, *p < 0.05, **p < 0.01, ***p < 0.001, ****p < 0.0001.

A significant increase in mean mPAP from 15.6 ± 3.5 mmHg at the time point of first intervention (Week 1) to 35 ± 7.7 mmHg (*p* = 0.005) at Week 6 (Figure 3) and a significant increase in mean total pulmonary resistance (TPR) from 3.4 ± 0.9 WU to 6.2 ± 2.7 WU (*p* = 0.043) was observed (Figure 3). PAWP could be measured in *n* = 14/30, 46.7% across the study and in *n* = 0/5, 0% at Week 6. The mean PVR, calculated only when PAWP was available, increased from 1.06 WU at Week 1 to 4.53 WU at Week 5. However, due to sparse paired PAWP availability, especially the absence of Week 6 PAWP measurements, this comparison should be interpreted descriptively rather than as a robust paired longitudinal test (Supporting Figure 1).

### 3.2 MRI – Right Ventricular Changes

**Figure 4:**
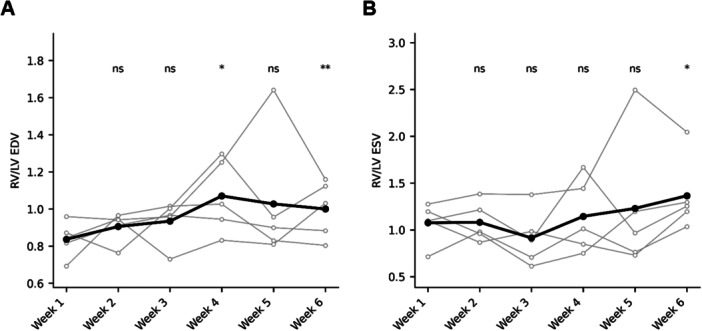
Right ventricular growth normalized to the left ventricular growth, reflecting the normal non‐affected heart growth of the animal. A significant increase in right ventricular enddiastolic (RV/LV EDV Ratio, *p* = 0.0038) (A) and endsystolic volume (RV/LV ESV Ratio, *p* = 0.042) (B) were observed. Individual changes are shown from Week 1 to Week 6, with gray lines representing individual pigs and the thicker black line representing the group mean. Statistical comparisons were performed for each week versus Week 1 using paired *t*‐tests. Significance annotations indicate ns, not significant, *p < 0.05, **p < 0.01, ***p < 0.001, ****p < 0.0001.

**Figure 5:**
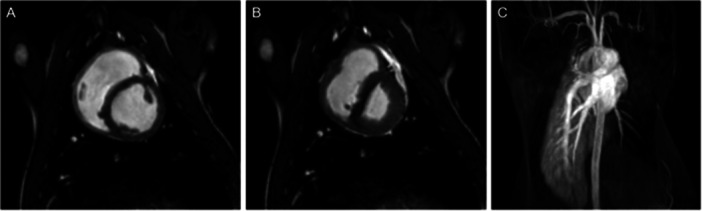
Functional cardiovascular MRI studies performed prior to sacrifice (Week 6) showing the dilatation of the right ventricle (A), flattening of the intraventricular septum towards the left ventricle (B) and complete perfusion defect of the left lung through the left pulmonary artery plug occlusion (C).

Comparing the right ventricular ejection fraction between Week 1 and Week 6, no significant change was observed (*p* = 0.127), instead there was a significant increase in right ventricular stroke volume (*p* = 0.0017), right ventricular end‐diastolic (*p* = 0.00061) and end‐systolic volume (*p* = 0.0098) (Figure 3, Supporting Table 1). When normalizing the right ventricular growth to the left ventricular growth, which reflects the normal non‐affected heart growth of the animal, a significant increase in right ventricular end‐diastolic (*p* = 0.0038) and end‐systolic volume (*p* = 0.042) were observed (Figure 4). In the MRI images, the dilatation of the right ventricle was also visible, and some animals showed a bowing or flattening of the intraventricular septum towards the left ventricle (Figure 5).

### 3.3 MRI – Left Ventricular Changes

The left ventricular cardiac output did not significantly change over the experimental course of 5 weeks (*p* = 0.066). There was a significant increase in left ventricular ejection fraction (*p* = 0.0256), stroke volume (*p* = 0.0022) and left ventricular end‐diastolic volume (*p* = 0.0067) but no significant change in end‐systolic volume (*p* = 0.0719) (Supporting Table 1).

### 3.4 Macroscopic Assessment of Harvested Lungs

**Figure 6:**
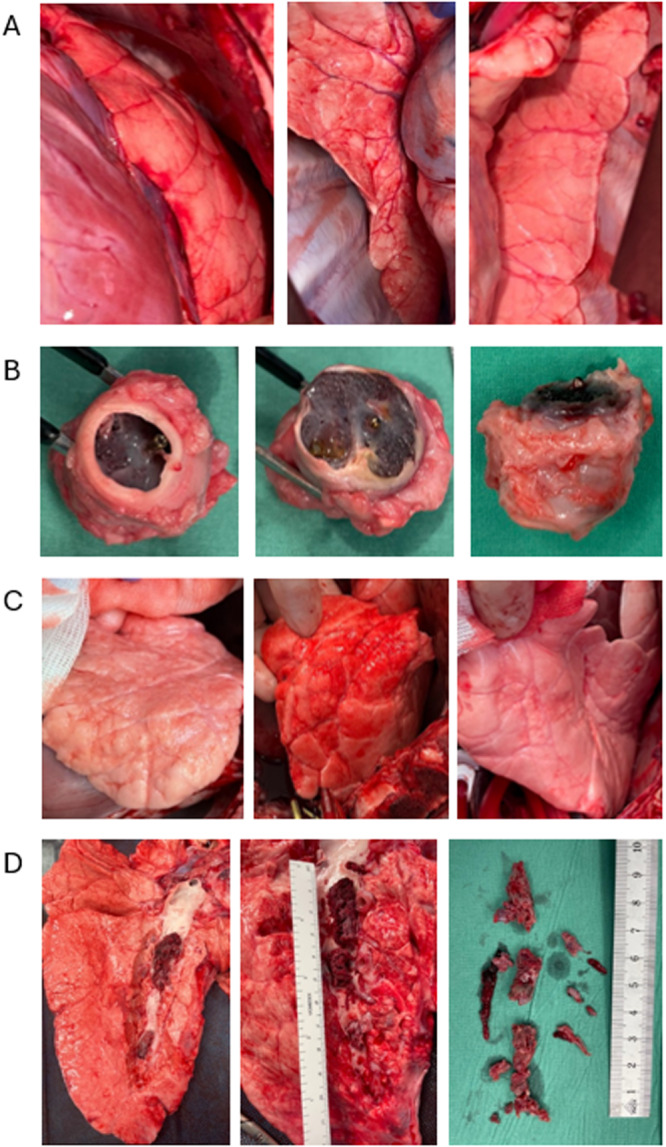
Macroscopic changes of the lungs and pulmonary arteries. (A) Hypertrophic collateral bronchial circulation of the left lung, (B) Intravascular plug fully closing the left PA and fully ingrown within the left PA wall. (C) Overflow region with edema in the right upper lobe. (D) Glue‐thrombus evacuated in the lower right lobe artery.

In all pigs, distinct areas of ischemia with hypertrophy and an increase in bronchial arteries on the left lung, where the intravascular plug occluded the left pulmonary artery, developed. On the right side, a hyperperfused area in the right upper lobe and areas of ischemia and atelectasis on the right lower lobe developed, in which the residual glue‐thrombus from the right lower lobe artery (Figure 6) was evacuated. No other macroscopic abnormalities were observed.

### 3.5 Secondary Microvasculopathy Development

**Figure 7:**
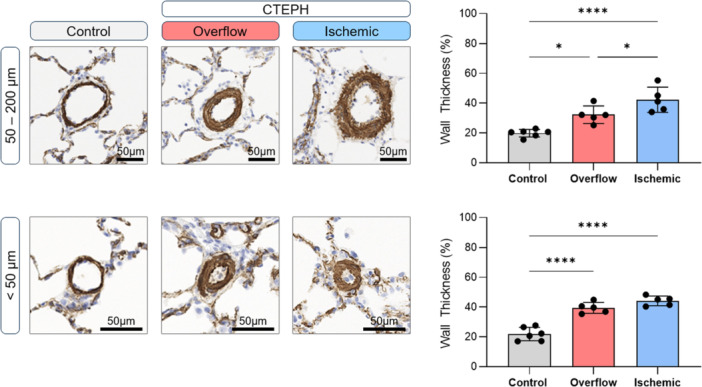
Quantification of vascular wall thickness on α‐SMA distal lung staining from control tissue, the right upper lobe (overflow territory), and the left upper lobe (ischemic territory). Quantification was performed in small pulmonary arteries (< 50 µm) and intermediate‐sized pulmonary arteries (50–200 µm). Wall thickness is expressed as percentage (%). Significance annotations indicate *p < 0.05, **p < 0.01, ***p < 0.001, ****p < 0.0001.

Histological analysis stratified by vessel diameter confirmed secondary pulmonary microvascular remodeling in both obstructed and overflow territories. In small pulmonary arteries (< 50 µm), wall thickness was increased in ischemic obstructed, and overflow non‐obstructed regions compared with healthy control tissue (*p* < 0.0001). Similar changes were observed in intermediate‐sized arteries (50–200 µm) (*p* < 0.0001). These findings show that vascular remodeling developed in both chronically ischemic and hyperperfused lung territories, consistent with the secondary vasculopathy observed in human CTEPH (Figure 7).

### 3.6 Assessment of Key Vasoactive Pathways During PH Development

**Figure 8:**
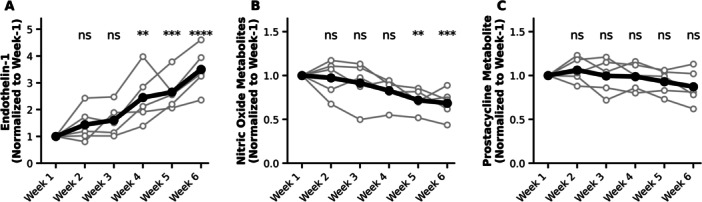
Molecular pathways associated with CTEPH development. Plasma analysis in pigs from Week 1 (CTEPH induction) to Week 6 for (A) endothelin 1 (≈ 3.5fold increase at week‐6), (B) nitric oxide metabolites (≈ 0.7fold decrease at week‐6), and (C) the prostacyclin metabolite.

Circulating endothelin 1 levels significantly and progressively increased (≈ 3.5‐fold at Week 6) (*p* < 0.0001) during PH development over time. Furthermore, circulating nitric oxide metabolites decreased (≈ 0.7‐fold at Week 6) (*p* = 0.0007). However, no significant change was detected in circulating prostacyclin metabolite levels (*p* = 0.232) (Figure 8).

## 4 Discussion

This porcine CTEPH model represents a refined version of a previously described CTEPH model. Importantly, this study represents a procedural refinement of the established piglet CTEPH model [7]. The principal gain is the avoidance of sternotomy or direct surgical pulmonary artery ligation, which likely reduces perioperative trauma, recovery burden, and procedural complexity. Conversely, the present study does not yet provide the same depth of mechanistic phenotyping reported in earlier model series, particularly regarding RV reserve, exercise or pharmacologic stress testing. The increase in mPAP together with increased TPR and, where available, increased PVR supports the development of a CTEPH phenotype [2]. MRI assessments revealed significant RV remodeling, with increased RV stroke volume and end‐diastolic and end‐systolic volumes, indicative of the RV adaptation to the increased pulmonary vascular resistance. These hemodynamic and MRI findings are consistent with the development of RV failure, a key feature of CTEPH, further validating the model's ability to replicate the disease's pathophysiology [12]. Additionally, using MRI allowed non‐invasive monitoring of right ventricular changes, such as dilatation and intraventricular septal flattening, which are characteristic of CTEPH‐induced RV failure [2, 12]. In the harvested lungs, distinct macroscopic features of CTEPH, consistent with those found in human patients, developed [13, 14]. The left lung, where the intravascular plug occluded the left PA, displayed areas of ischemia and hypertrophic bronchial collateral arteries. On the right side, overperfusion in the right upper lobe and ischemia in the right lower lobe were observed, the latter caused by the repeated glue embolization in this region. These macroscopic changes reflect the altered pulmonary blood flow patterns characteristic of CTEPH, where blood is diverted away from obstructed areas, causing overperfusion in non‐affected regions and ischemia in obstructed territories [2, 13, 14]. Another major component of CTEPH pathophysiology is the development of secondary microvasculopathy, which can arise after obstruction in ischemic territories but also in overperfused non‐obstructed territories [7, 13, 15]. In this refined model, the diameter‐stratified analysis of pulmonary arteries showed that pulmonary arterial remodeling was present in both very small arteries (< 50 µm) and larger small arteries (50–200 µm), as described in the original model [8]. This supports the presence of distal arterial remodeling across different arterial calibers and both obstructed and overflow territories. Therefore, the present histology demonstrates an important component of secondary microvasculopathy.

Currently, precapillary PH is managed with therapies that target three vasoactive pathways: endothelin‐1, nitric oxide or prostacyclin pathways [11]. Endothelin‐1 signaling was shown to be increased in the previous piglet model [8]. In this refined model, not only an increase in circulating endothelin‐1 associated with the development of PH is seen, but also a decrease in nitric oxide metabolites. The recapitulation of selected macroscopic and microscopic features and key alterations in vasoactive pathways in this model supports its potential physiological relevance and underscores its value for studying disease mechanisms, future identification and evaluation of novel therapeutic targets and studying potential therapeutic interventions.

The development of animal models that accurately replicate the complex pathophysiology of CTEPH has been challenging, and current CTEPH large and small animal models come with inherent limitations [3, 4]. In small‐animal models, while offering practical and ethical advantages, the small lung and vessel size make it difficult to reproduce human‐like proximal segmental and lobar lesions, or to capture the spatial heterogeneity of perfusion, large‐vessel flow patterns, and extensive bronchial‐collateral networks and the complex vascular architecture relevant for surgical or interventional strategies, such as PEA and BPA [3, 4, 5, 15, 16, 17]. Therefore, large‐animal CTEPH models are indispensable for reproducing human‐like cardiopulmonary anatomy, hemodynamics, and RV‐adaptation to chronic thromboembolic obstruction [3, 4]. Large‐animal models reliably produce chronic precapillary PH with sustained elevations in mPAP and TPR or PVR, along with RV hypertrophy and impaired RV function [9, 18, 19, 20, 21]. Limitations of large animal models are their invasive nature, requiring surgical procedures that pose significant risks, including infection, prolonged recovery periods, and animal suffering [22]. These constraints may hinder their broader development, implementation, and adoption across research teams. Other limitations are high cost, logistical complexity, ethical constraints, and sometimes limited distal vascular and RV histology mimicking. Distal microvasculopathy is variably developed and often requires additional insults or remains less well characterized than in human disease [3, 4, 17].

The porcine model presented here addresses several practical limitations of surgical large‐animal CTEPH models by accurately reproducing major hemodynamic characteristics, regional perfusion changes, macrovascular characteristics, wall thickening of small pulmonary arteries and known molecular changes of human CTEPH. Additionally, the refined model minimizes invasiveness by replacing the open‐chest surgery with a transcatheter intravascular plug placement. While eliminating the need for open surgery, this approach reduces surgical trauma and potentially improves the overall welfare of the animals. Despite the less invasive intervention, the model successfully reproduces critical hemodynamic changes seen in CTEPH, such as increased mPAP and TPR, as well as significant RV remodeling, including increased stroke volume and RV end‐diastolic and end‐systolic volumes. These findings highlight that the model preserves the essential features of CTEPH while reducing invasiveness and increasing safety. Consequently, the presented refined model demonstrates a commitment to the 3Rs principles, particularly the “refinement” and “reduction” aspects [10]. No animal died during the induction of a CTEPH condition. By reducing the need for major surgical procedures, premature animal death and stress, and the number of animals required for longitudinal studies can potentially be reduced, and the minimally invasive approach may facilitate wider adoption of the model. However, the current study should be viewed as a feasibility and refinement study, and additional mechanistic work will be required to define the full physiological and histopathological spectrum of the model.

Concurrent work was recently published establishing a minimally invasive approach using repeated injections of silk sutures into both left and right pulmonary arteries [20]. In direct comparison, the advantages of our model lie in the finely separated vascular territories with spatially structured disease with clearly defined obstructed, overperfused, and reduced‐flow lung regions, which are powerful for studying regional bronchial collateralization, microvasculopathy and flow redistribution. Additionally, it is refining a pre‐existing, well‐validated and characterized reference CTEPH large‐animal model, supporting further reproducibility and validation of the model [3, 4, 7, 9].

In summary, while current CTEPH models have contributed significantly to improved understanding of the disease, the presented minimally invasive porcine model provides a more ethical and promising refinement of an established porcine CTEPH model and appears suitable for future studies focused on regional flow redistribution, bronchial collateralization, microvascular remodeling, and RV adaptation. Its reproducibility, robustness, and sensitivity to therapeutic interventions should be confirmed in larger cohorts with sham controls.

While the model provides valuable insights into CTEPH, there are several limitations. First, the sample size was relatively small and did not include a contemporary sham catheterization or sham embolization group. This limits conclusions regarding reproducibility, generalizability and model‐specific longitudinal changes. Additionally, all pigs included in the study were female, as male experimental animals are routinely castrated; this may further limit the applicability of the results to broader pig populations. The relatively short duration of the study (6 weeks) may also limit the understanding of the long‐term effects of CTEPH and the potential for recovery following therapeutic interventions. Furthermore, PAWP was not measurable in every animal and at every time point, reflecting technical limitations of repeated large‐animal catheterization under the experimental conditions. Incomplete PAWP measurements limited PVR calculation to a subset of measurements. TPR was therefore retained as the primary resistance metric, while PVR is reported only where PAWP was available. Additionally, bronchial arterial hypertrophy was assessed macroscopically in the present study. A systematic microscopic quantification of peribronchial systemic vessels and vasa vasorum was not performed and should be included in future studies.

In conclusion, this study demonstrates the feasibility of an animal welfare‐oriented refined, minimally invasive porcine CTEPH model that reproduces key hemodynamic, morphological, and molecular features of the disease previously described in a surgical model while avoiding open‐chest surgery. By closely mimicking human CTEPH in a fully minimally invasive manner, this model provides a valuable tool for investigating disease mechanisms and evaluating novel therapeutic approaches in a way that prioritizes animal welfare. The refined model appears well‐suited for longitudinal assessment of regional perfusion redistribution and RV adaptation, but its robustness and mechanistic breadth require confirmation in larger studies.

## Author Contributions

Conception and design: Miriam Weisskopf, Isabelle Opitz, Bianca Battilana. Administrative support: all authors. Provision of study materials or patients: all authors. Collection and assembly of data: all authors. Data analysis and interpretation: all authors. Manuscript writing: all authors. Final approval of manuscript: all authors.

## Conflicts of Interest

Bianca Battilana: No real conflict of interest in relation to this manuscript. She received funding for protected research time from the Swiss National Science Foundation under the IMPACT project (IMproved diagnosis and Personalized treAtment for patients with Chronic Thromboembolic Pulmonary Hypertension) and the European Society for Thoracic Surgeons (ESTS) Biology Club Research Fellowship Award 2024.

Christian Stoeck: No conflict of interest in relation to this manuscript.

Fabien Robert: No conflict of interest in relation to this manuscript.

Ömer Senbaklavaci: No conflict of interest in relation to this manuscript.

Miriam Weisskopf: No conflict of interest in relation to this manuscript.

Isabelle Opitz: No real conflicts of interest. The following could be perceived as such: Roche (Institutional Grant), AstraZeneca (Advisory Board and Steering Committee), MSD (Advisory Board), BMS (Advisory Board), Medtronic (Institutional Grant and Advisory Board), Intuitive (Proctorship and Speakers Fee), Sanofi (Speakers Fee), Regeneron (Advisory Board), XVIVO (Institutional Grant), Siemens (Speakers Fee), Astellas (Speakers Fee). I.O. is IASLC Board Director, Member of the Thoracic Clinical Practice Standards Committee and the Thoracic Education Committee of AATS, iMig Board Member and JTCVS Associate Editor. She is in the Stiftungsrat Schulthessklinik and an Advisory Board Member at Med Uni Wien for Comprehensive Center for Chest Diseases (CCCD).

## Supporting information

Supporting File 1

Supporting File 2

## Data Availability

The data that support the findings of this study are available from the corresponding author upon reasonable request. The data underlying this article will be shared on reasonable request to the corresponding author.
