# Supplementary material for: Minimally Invasive Porcine Model for Chronic Thromboembolic Pulmonary Hypertension
Source: Pulm Circ. 2026 Jul 14;16(3):e70344. doi: 10.1002/pul2.70344 (PMC13366112; doi:10.1002/pul2.70344)
Supplement: Supplementary file 1 — Supporting File 1 [file PUL2-16-e70344-s001.docx]

**Supplementary Figures:**

| **A**  **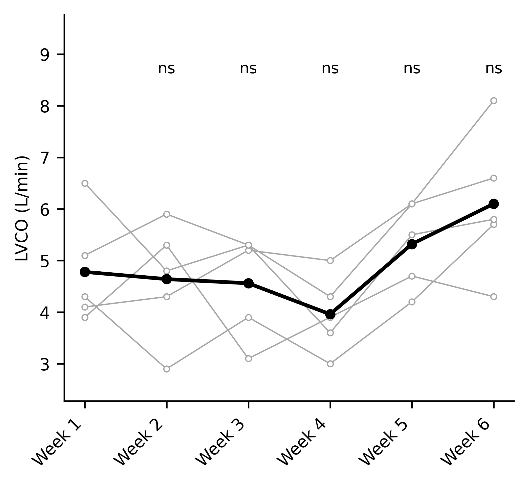** | **B**  **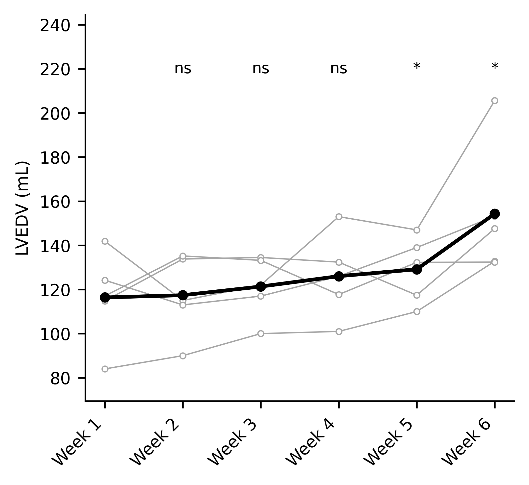** |
| --- | --- |
| **C**  **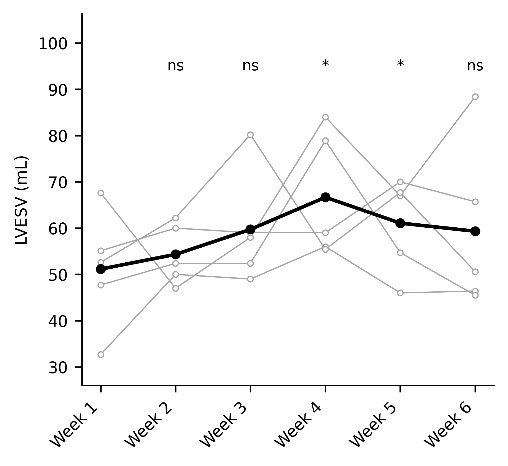** | **D**  **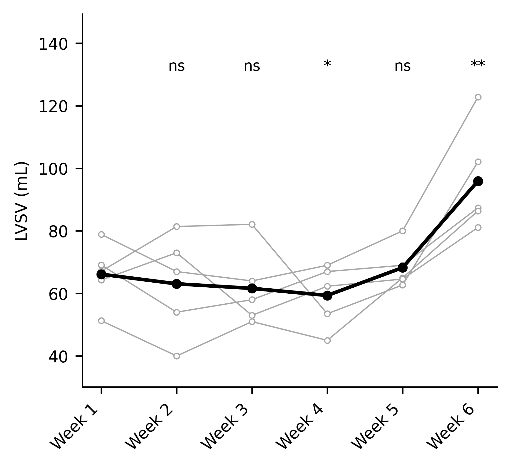** |
| **E**  **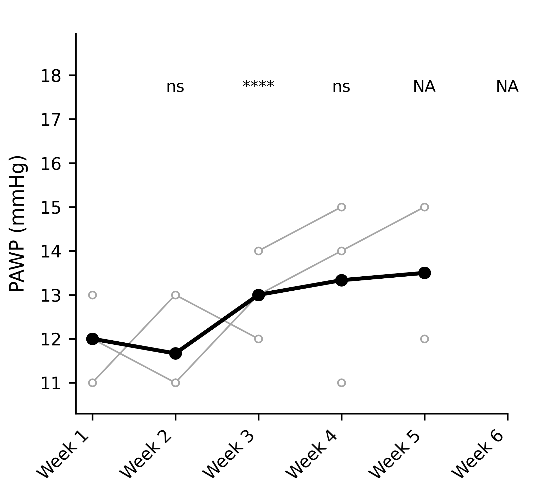** | **F**  **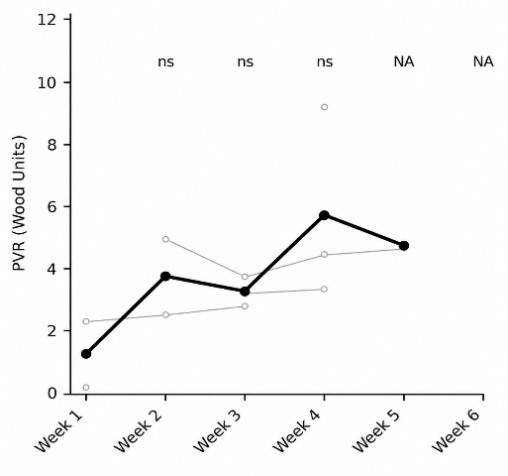** |

**Supplementary Figure 1:** Panels show **(A)** left ventricular cardiac output (LVCO), **(B)** left ventricular end-diastolic volume (LVEDV), **(C)** left ventricular end-systolic volume (LVESV), **(D)** left ventricular stroke volume (LVSV), **(E)** pulmonary arterial wedge pressure (PAWP) when measurable, and **(F)** pulmonary vascular resistance (PVR) for the values with available PAWP. Individual changes are shown from Week 1 to Week 6, with grey lines representing individual pigs and the thicker black line representing the group mean. Statistical comparisons were performed for each week versus Week 1 using paired t-tests. Significance annotations indicate ns, not significant, * p < 0.05, ** p < 0.01, *** p < 0.001, **** p < 0.0001. NA indicates that statistical comparison was not possible because of insufficient paired measurements.
