# Supplementary material for: Minimally Invasive Porcine Model for Chronic Thromboembolic Pulmonary Hypertension
Source: Pulm Circ. 2026 Jul 14;16(3):e70344. doi: 10.1002/pul2.70344 (PMC13366112; doi:10.1002/pul2.70344)
Supplement: Supplementary file 2 — Supporting File 2 [file PUL2-16-e70344-s002.docx]

**Supplementary Material**

**Supplementary Table 1:** Hemodynamic parameters measured with a Right heart catheter (Swan-Ganz) and functional heart MRI at Week 1 (baseline, induction of CTEPH) and Week 6 (prior to sacrifice). Values are reported as means ± standard deviation. Differences were calculated with a paired t-test.

|  | **Week 1** | **Week 6** | **p-value** |
| --- | --- | --- | --- |
| Mean pulmonary artery pressure (mPAP), mmHg | 15.6 ±3.5 | 35 ±7.7 | **0.005** |
| Total pulmonary resistance (TPR), WU | 3.4 ±0.9 | 6.2 ±2.7 | **0.04** |
| Right ventricular ejection fraction (RVEF), % | 44.8 ±3.6 | 49.4 ±6 | 0.127 |
| Right ventricular stroke volume (RVSV), ml | 43.26 ±6.6 | 75.08 ±9.6 | **0.0017** |
| Right ventricular enddiastolic volume (RVEDV), ml | 96.52 ±13.4 | 152.34 ±15.5 | **0.00061** |
| Right ventricular endsystolic volume (RVESV), ml | 53.26 ±8.4 | 77.28 ±13.7 | **0.0098** |
| Left ventricular cardiac output (LVCO), ml | 4.78 ±1 | 6.1 ±1.2 | 0.066 |
| Left ventricular ejection fraction (LVEF), % | 57.2 ±2.1 | 62.2 ±4.4 | **0.0256** |
| Left ventricular stroke volume (LVSV), ml | 66.1 ±8.9 | 96.1 ±15 | **0.0022** |
| Left ventricular enddiastolic volume (LVEDV), ml | 116.38 ±18.8 | 155.44 ±29 | **0.0067** |
| Left ventricular endsystolic volume (LVESV), ml | 51.1 ±11.3 | 59.32 ±16.2 | **0.0719** |
| Right ventricular over left ventricular enddiastolic volume (RV/LV EDV) Ratio | 0.83 ±0.09 | 1 ±0.14 | **0.0038** |
| Right ventricular over left ventricular endsystolic volume (RV/LV ESV) Ratio | 1.07 ±0.19 | 1.4 ±0.35 | **0.042** |
